# Eight-and-a-half syndrome as the first presentation of multiple sclerosis in an Asian male: a case report

**DOI:** 10.1186/s13256-022-03699-8

**Published:** 2023-03-06

**Authors:** Xin Ying Lim, Yong Zheng Wai, Yun Xiu Yong, Lik Thai Lim

**Affiliations:** 1 Hospital Queen Elizabeth, Kota Kinabalu, Sabah Malaysia; 2 Universiti Malaysia Sarawak (UNIMAS), Kota Samarahan, Sarawak Malaysia

**Keywords:** Eight-and-a-half syndrome, Multiple sclerosis, Asian, Case report

## Abstract

**Background:**

Multiple sclerosis is a diffuse chronic demyelinating disease of the central nervous system. It is relatively uncommon in the Asian population and even more so in males. Despite the usual involvement of the brainstem, eight-and-a-half syndrome remains a rare first presentation in multiple sclerosis. Only a few cases have been reported previously, but none involving the Asian population. Eight-and-a-half syndrome, a neuro-ophthalmological condition, is characterized by one-and-a-half syndrome with ipsilateral lower facial nerve palsy, which localizes lesions to the pontine tegmentum. This case report demonstrates the first case of eight-and-a-half syndrome as the first presentation of multiple sclerosis in an Asian male.

**Case presentation:**

A healthy 23-year-old Asian man presented with sudden onset of diplopia followed by left-sided facial asymmetry for 3 days. Assessment of extraocular movement revealed left conjugate horizontal gaze palsy. On right gaze, there was limited left eye adduction and horizontal nystagmus of the right eye. These findings were consistent with a left-sided one-and-a-half syndrome. Prism cover test revealed left esotropia of 30 prism diopters. Cranial nerve examination showed left lower motor neuron facial nerve palsy, while other neurological examination was normal. Magnetic resonance imaging brain showed multifocal T2 fluid attenuated inversion recovery hyperintense lesions, involving bilateral periventricular, juxtacortical, and infratentorial regions. A focal gadolinium contrast-enhanced lesion with open ring sign on T1 sequence was seen at the left frontal juxtacortical region. Multiple sclerosis was diagnosed on the basis of the clinical and radiological evidence, which fulfilled the 2017 McDonald criteria. Positive oligoclonal bands in cerebrospinal fluid analysis further confirmed our diagnosis. He had a complete resolution of symptoms 1 month after a course of pulsed corticosteroid therapy, and was subsequently placed on maintenance therapy with interferon beta-1a.

**Conclusion:**

This case illustrates eight-and-a-half syndrome as the first presentation of a diffuse central nervous system pathology. A wide range of differential diagnoses needs to be considered in such a presentation as based on the patient’s demographics and risk factors.

## Background

Multiple sclerosis (MS) is a chronic disease of the central nervous system (CNS) characterized by loss of motor or sensory function due to immune-mediated inflammation, demyelination, and subsequent loss of axons [1]. It is relatively rare in Asia, with a prevalence of 2–5/100,000 and a female-to-male ratio of 4:1[1]. Despite known association of brainstem signs with MS [2], eight-and-a-half syndrome remains a rare first presentation. There were a few cases reported previously, but none among the Asian population. Eight-and-a-half syndrome is a neuro-ophthalmological condition characterized by one-and-a-half syndrome and ipsilateral lower facial nerve palsy, which was first described in 1998 by Eggenberger [3]. This case report demonstrated the first case of eight-and-a-half syndrome as the first presentation of MS in an Asian male.

## Case presentation

**Fig. 1:**
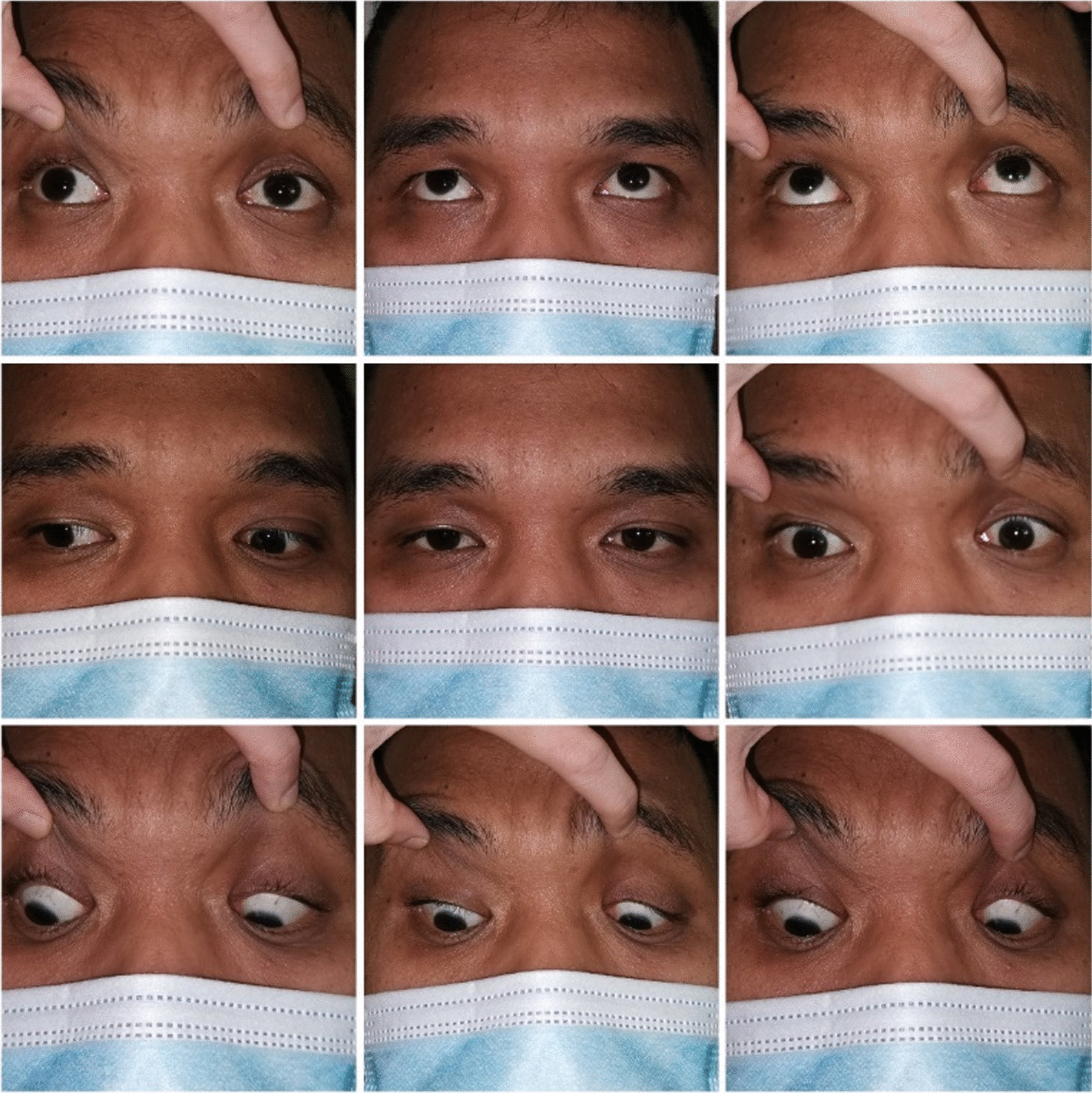
Left gaze palsy in the nine diagnostic positions of gaze. Upon right gaze, left eye was unable to adduct past midline with right eye horizontal nystagmus upon abduction

**Fig. 2:**
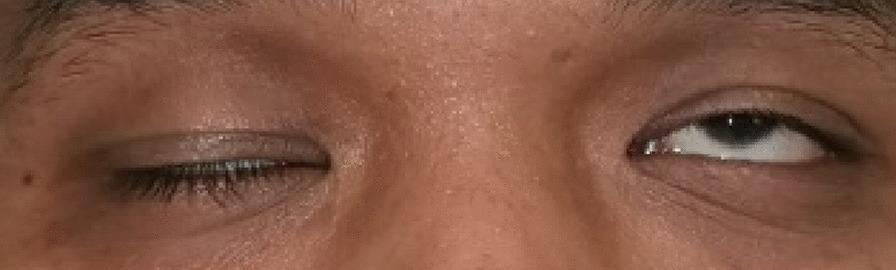
Left lower motor facial nerve palsy demonstrated by mild left lagophthalmos and loss of nasolabial folds

A healthy 23-year-old Asian man presented with sudden onset of diplopia followed by left-sided facial asymmetry for 3 days. On examination, he appeared well orientated without impaired cognition. Visual acuity and color vision was normal. Bilateral pupils were 3 mm equal and reactive to light with an absent relative afferent pupillary defect. Assessment of extraocular movement revealed a complete left conjugate horizontal gaze palsy. Upon right gaze, there was limited left eye adduction and horizontal nystagmus of the right eye upon abduction (Fig. 1). The impaired extraocular movement was not corrected by oculocephalic reflex. Prism cover test (PCT) revealed left esotropia of 30 prism diopters (PD). There was no ptosis and convergence was normal. Fundus examination was unremarkable. Cranial nerve examination showed left lower motor neuron facial nerve palsy, as demonstrated by the inability to wrinkle left frontalis, left lagophthalmos, and shallow nasolabial folds (Fig. 2). Other neurological examinations were normal.

**Fig. 3:**
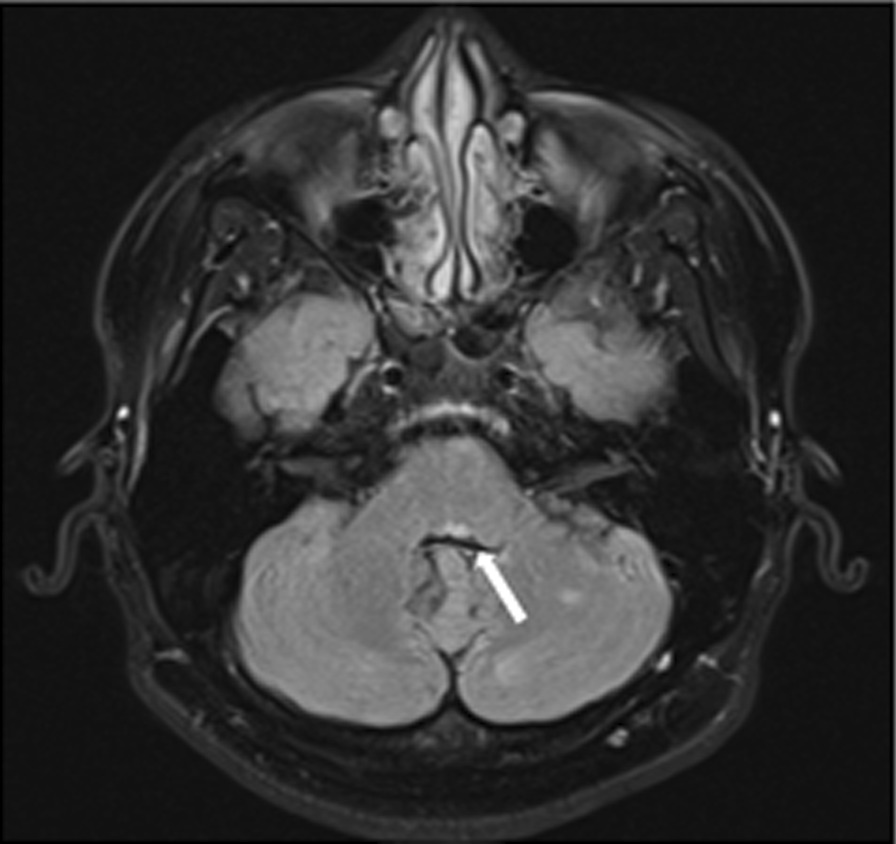
MRI brain T2 FLAIR showing hyperintense lesion (white arrow) over left pontine tegmentum causing left eight-and-a-half syndrome in this patient

Brain magnetic resonance imaging (MRI) was done and showed multifocal T2 fluid attenuated inversion recovery (FLAIR) hyperintense lesions without restricted diffusion, involving bilateral periventricular, juxtacortical, and infratentorial regions, which includes left pontine tegmentum (Fig. 3). A focal gadolinium-enhanced lesion with an open ring sign was seen at left frontal juxtacortical. A diagnosis of MS was made on our patient’s first presentation based on his MRI brain findings, which fulfilled the latest McDonald criteria (2017). Cerebrospinal fluid (CSF) investigation revealed positive oligoclonal bands, which further confirmed our diagnosis. Pulsed steroid therapy was commenced, and his symptoms completely resolved after 1 month. He was subsequently placed on maintenance therapy with interferon beta-1a and followed up by our neuro-medical team.

## Discussion and conclusions

Horizontal gaze is regulated by parapontine reticular formation (PPRF), medial longitudinal fasciculus (MLF), abducens, and oculomotor nuclei. PPRF receives impulse from contralateral frontal eye field (FEF) and ipsilateral parietal cortex, acting as the last supranuclear relay involved in conjugate horizontal eye movement. The impulse is then relayed to ipsilateral abducens nucleus and via the MLF to contralateral oculomotor nucleus, causing contraction of ipsilateral lateral rectus and contralateral medial rectus muscle, resulting in horizontal gaze.

One-and-a-half syndrome [4] is a combination of internuclear ophthalmoplegia (INO) and horizontal gaze palsy. INO is caused by lesions in the MLF, while horizontal gaze palsy may be due to lesions in PPRF, abducens nucleus, or fascicles. Since our patient demonstrated an impaired oculocephalic reflex, we presumed that the lesion involved abducens nucleus or fascicles, rather than an isolated PPRF [3].

**Fig. 4:**
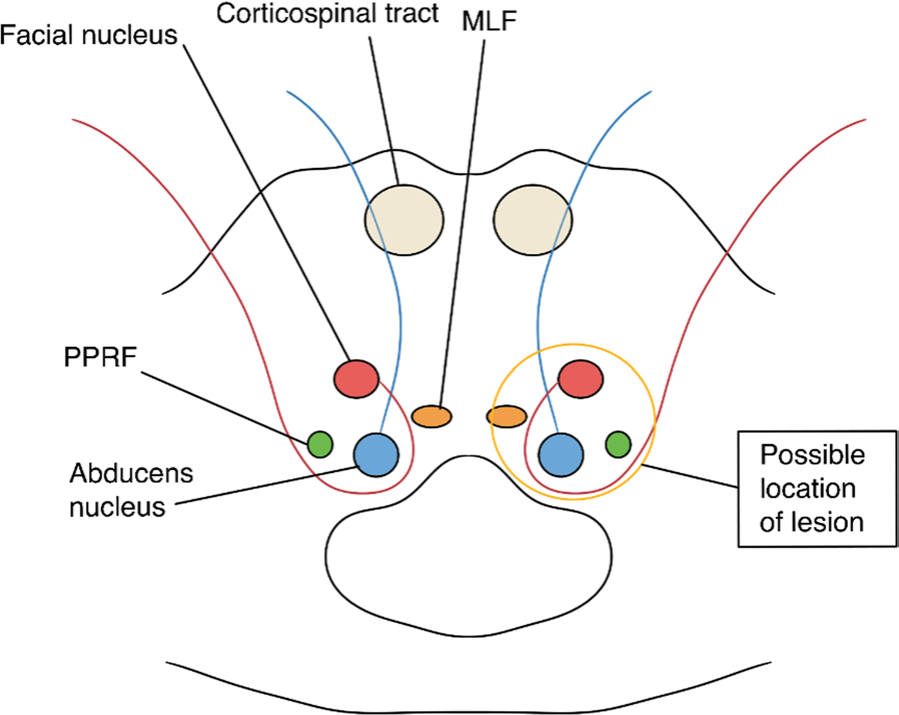
Schematic axial diagram of pontine tegmentum at the level of facial colliculus. The circled area represents the location of lesion in eight-and-a-half syndrome involving the left facial nucleus and its fascicle, in addition to the left horizontal gaze center structures, median longitudinal fasciculus (MLF), parapontine reticular formation (PPRF), abducens nucleus, and nerve

Rarely when the lesion extends to the ipsilateral facial nerve nucleus or its fascicles, which wrap around the abducens nucleus, does it result in ipsilateral lower motor neuron facial nerve palsy. The combination of both one-and-a-half syndrome (1.5) and lower motor neuron facial nerve palsy (7) is therefore termed as eight-and-a-half syndrome [5] (8.5). This syndrome localizes lesions to pontine tegmentum (as shown in Fig. 4), which houses horizontal gaze center structures, facial nucleus, and fascicles.

Among reported cases of eight-and-a-half syndrome, the most common etiology is ischemic stroke [3, 6], followed by intracranial hemorrhage [7], demyelinating lesion [3, 6], infection (tuberculoma) [6], and vasculitis [3]. Despite brainstem lesions in MS commonly accounting for 20% of clinical isolated syndromes [2], eight-and-a-half syndrome is still a rare first presentation. To date, there have only been a few cases reported, including one case of childhood MS [8]. Due to the rarity of MS among the Asian population, it is usually not among the top differential diagnoses for clinicians.

With the advancement of neuroimaging, clinical evidence of dissociation in space and time in MS can be demonstrated radiologically with the revised 2017 McDonald criteria [9], allowing for the possible diagnosis of MS on the first clinical presentation.Our patient’s clinical isolated syndrome lesion was shown on MRI as T2/FLAIR hyperintensity over the left pontine tegmentum. Other asymptomatic T2/FLAIR hyperintense lesions were found at typical locations for MS; namely, juxtacortical, periventricular, and infratentorial regions, which illustrate dissociation in space. Dissociation in time is evidenced by the presence of gadolinium-enhanced T1 lesion at the left frontal subcortical region with other concurrent non-enhancing lesions. It is therefore sufficient to diagnose our patient with MS on his first presentation, which was further supported by positive CSF oligoclonal bands.

To the best of our knowledge, this case demonstrated the first case of eight-and-a-half syndrome as the first sign of MS in an Asian male. Eight-and-a-half syndrome remains a relatively rare brainstem lesion. Despite localization of the lesion to pontine tegmentum, it can still present as a clinical isolated syndrome of diffuse CNS disease. A wide range of differential diagnoses needs to be considered in such a presentation as based on the patient’s demographics and risk factors.

## Data Availability

The data and materials gathered during this study are included within the article.

## Abbreviations

MS: Multiple sclerosis
CNS: Central nervous system
MRI: Magnetic Resonance Imaging
CSF: Cerebrospinal fluid
PPRF: Parapontine reticular formation
MLF: Medial longitudinal fasciculus
FEF: Frontal eye field
INO: Internuclear ophthalmoplegia
